# Supplementary figures and images for: Systematic tissue-specific functional annotation of the human genome highlights immune-related DNA elements for late-onset Alzheimer’s disease
Source: PLoS Genet. 2017 Jul 24;13(7):e1006933. doi: 10.1371/journal.pgen.1006933 (PMC5546707; doi:10.1371/journal.pgen.1006933)

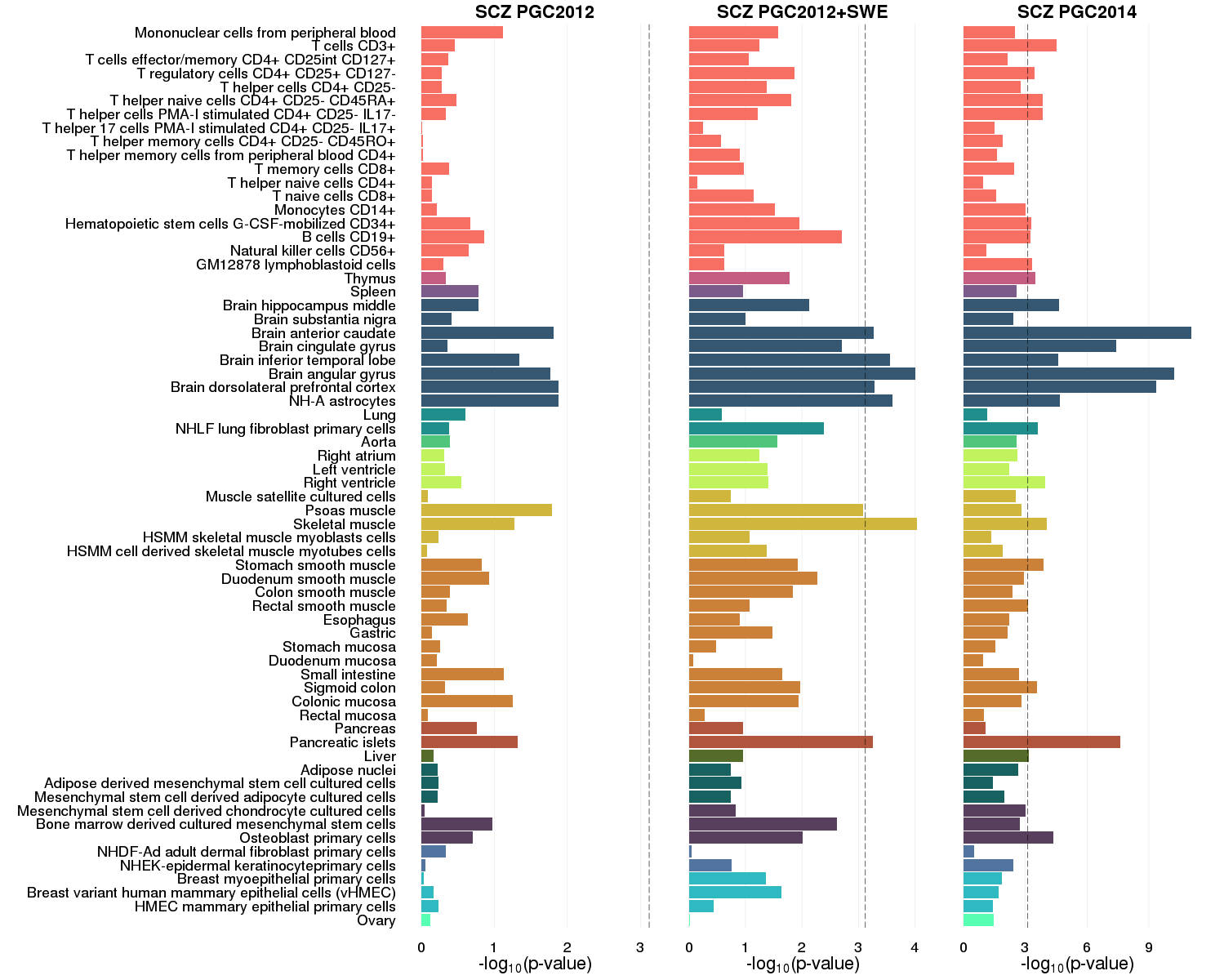

Supplement: S1 Fig — Overall enrichment pattern for schizophrenia remains stable as sample size increases. Sample sizes for the three studies shown below are 21,856, 32,143, and 82,315, respectively. (PNG) [file pgen.1006933.s001.png]

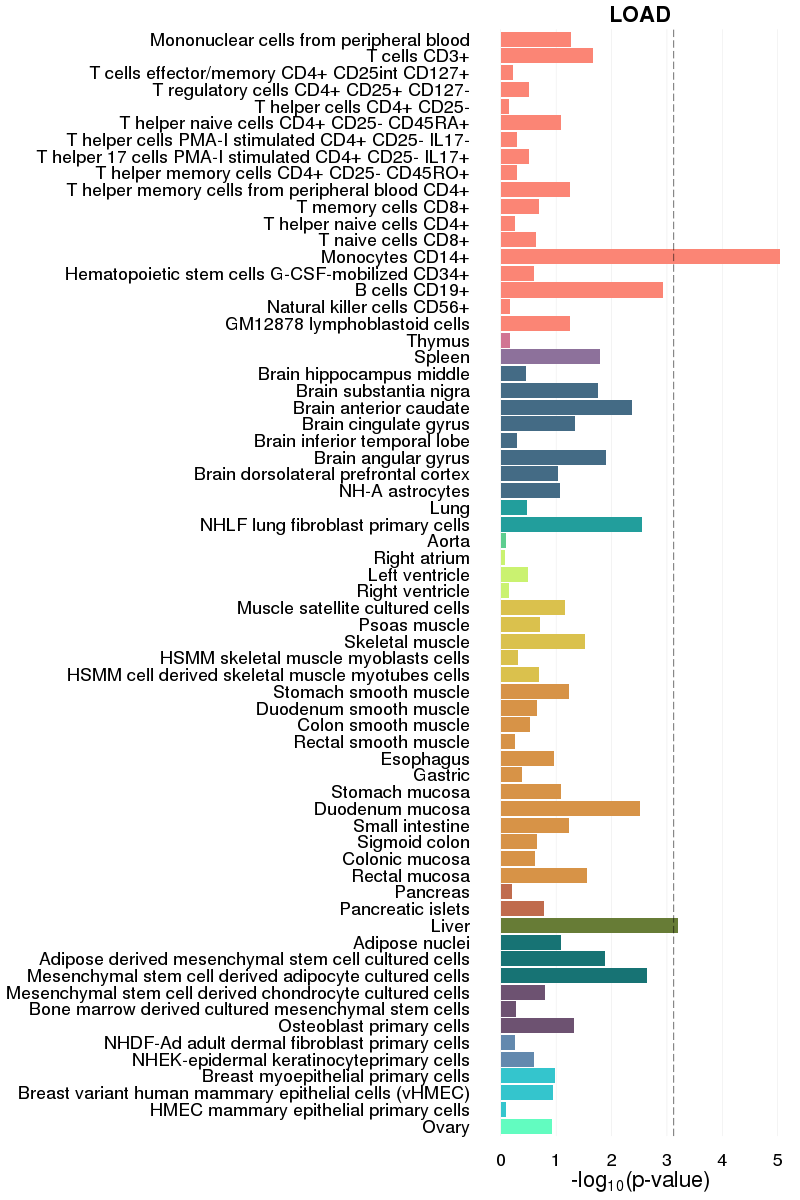

Supplement: S2 Fig — (PNG) [file pgen.1006933.s002.png]

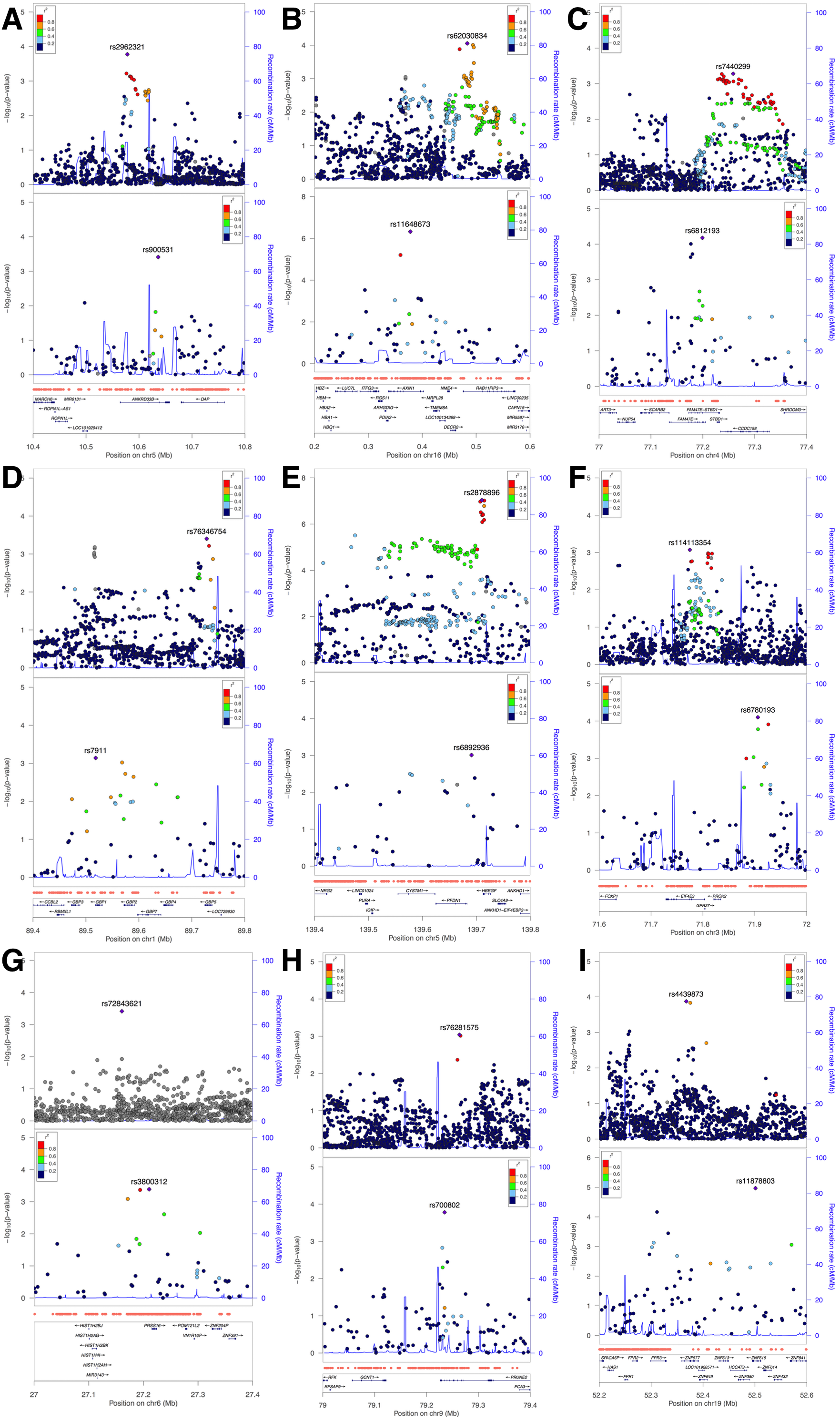

Supplement: S3 Fig — For each locus, the upper and lower panels show associations for LOAD and PD, respectively. Monocyte functional regions are marked by red dots above gene names. (PNG) [file pgen.1006933.s003.png]

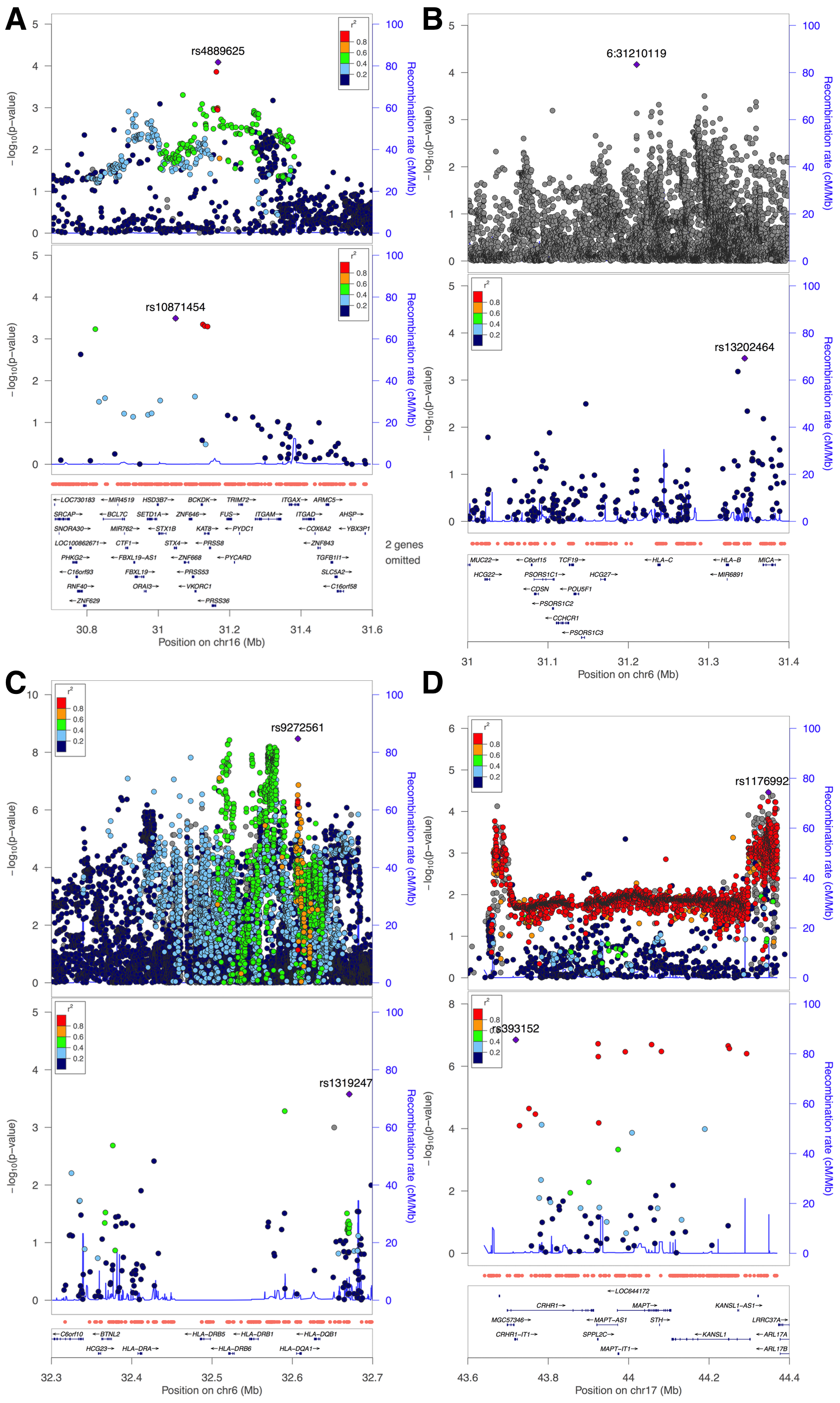

Supplement: S4 Fig — For each locus, the upper and lower panels show associations for LOAD and PD, respectively. Monocyte functional regions are marked by red dots above gene names. (PNG) [file pgen.1006933.s004.png]
