# Supplementary material for: Systematic tissue-specific functional annotation of the human genome highlights immune-related DNA elements for late-onset Alzheimer’s disease
Source: PLoS Genet. 2017 Jul 24;13(7):e1006933. doi: 10.1371/journal.pgen.1006933 (PMC5546707; doi:10.1371/journal.pgen.1006933)
Supplement: S3 Table — (PDF) [file pgen.1006933.s007.pdf]

S3 Table. Details of 45 genome-wide association studies.

Trait	Abbr.	Category	Sample Size*	Ref.	URL
Age at Menarche	AAM	Reproductive	132,989	[1]	http://www.reprogen.org/data_download.html
Age at Natural Menopause	AANM	Reproductive	69,360	[2]	http://www.reprogen.org/data_download.html
Asthma	AST	Immune/Pulmonary	26,475	[3]	http://www.cng.fr/gabriel/index.html
Eczema	ECZ	Immune	40,835	[4]	http://data.bris.ac.uk/data/dataset/28uchsdpmub118uex26ylacqm
Celiac Disease	CEL	Immune	15,283	[5]	https://www.immunobase.org
Crohn's Disease	CD	Immune/IBD	21,389	[6]	http://www.ibdgenetics.org
Ulcerative Colitis	UC	Immune/IBD	26,405	[7]	http://www.ibdgenetics.org
Primary Biliary Cirrhosis	PBC	Immune	13,239	[8]	https://www.immunobase.org
Rheumatoid Arthritis	RA	Immune	58,284	[9]	http://plaza.umin.ac.jp/~yokada/datasource/software.htm
Systemic Lupus Erythematosus	SLE	Immune	14,267	[10]	https://www.immunobase.org
Multiple Sclerosis	MS	Immune/Neurological	27,148	[11]	https://www.immunobase.org
Epilepsy	EPL	Neurological	34,853	[12]	http://www.epigad.org/page/show/homepage
Chronotype	CHT	Social/Psychiatric	127,898	[13]	http://www.t2diabetesgenes.org/data/
Subjective Well-being	SWB	Social/Psychiatric	298,420	[14]	http://www.thessgac.org/#!data/kuzq8
Depressive Symptoms	DEP	Social/Psychiatric	161,460	[14]	http://www.thessgac.org/#!data/kuzq8
Neuroticism	NEU	Social/Psychiatric	170,911	[14]	http://www.thessgac.org/#!data/kuzq8
Cognitive Performance	IQ	Neurological	106,736	[15]	http://www.thessgac.org/#!data/kuzq8
Education Years	EDU	Social	293,723	[16]	http://www.thessgac.org/#!data/kuzq8
Schizophrenia	SCZ	Psychiatric	82,315	[17]	https://www.med.unc.edu/pgc/results-and-downloads
Bipolar Disorder	BD	Psychiatric	16,731	[18]	https://www.med.unc.edu/pgc/results-and-downloads
Major Depressive Disorder	MDD	Psychiatric	18,759	[19]	https://www.med.unc.edu/pgc/results-and-downloads
Attention-Deficit/Hyperactivity Disorder	ADHD	Psychiatric	9,543	[20]	https://www.med.unc.edu/pgc/results-and-downloads
Anorexia Nervosa	AN	Psychiatric	14,477	[21]	https://www.med.unc.edu/pgc/results-and-downloads
Anxiety	ANX	Psychiatric	18,186	[22]	https://www.med.unc.edu/pgc/results-and-downloads
Ever Smoker	SMK	Psychiatric	74,053	[23]	https://www.med.unc.edu/pgc/results-and-downloads
Body Mass Index	BMI	Anthropometric	234,069	[24]	http://www.broadinstitute.org/collaboration/giant/index.php
Height	HT	Anthropometric	253,288	[25]	http://www.broadinstitute.org/collaboration/giant/index.php
Waist-hip Ratio (adjusted for BMI)	WHR	Anthropometric	142,762	[26]	http://www.broadinstitute.org/collaboration/giant/index.php
Systolic Blood Pressure	SBP	Cardiovascular	69,909	[27]	dbGap accession phs000585.v2.p1
Diastolic Blood Pressure	DBP	Cardiovascular	69,899	[27]	dbGap accession phs000585.v2.p1
Pulse Pressure	PP	Cardiovascular	74,079	[27]	dbGap accession phs000585.v2.p1
Birth Weight	BW	Growth	143,677	[28]	http://egg-consortium.org/birth-weight-2016.html
Fasting Glucose	GLU	Metabolic	58,074	[29]	http://www.magicinvestigators.org/downloads/
Fasting Insulin	INS	Metabolic	51,750	[29]	http://www.magicinvestigators.org/downloads/
Hemoglobin A1C	HBA	Metabolic	46,368	[30]	http://www.magicinvestigators.org/downloads/
High Density Lipoprotein	HDL	Metabolic	99,900	[31]	http://csg.sph.umich.edu/abecasis/public/lipids2010/
Low Density Lipoprotein	LDL	Metabolic	95,454	[31]	http://csg.sph.umich.edu/abecasis/public/lipids2010/
Total Cholesterol	TC	Metabolic	100,184	[31]	http://csg.sph.umich.edu/abecasis/public/lipids2010/
Triglyceride	TG	Metabolic	96,598	[31]	http://csg.sph.umich.edu/abecasis/public/lipids2010/
Coronary Artery Disease	CAD	Cardiovascular	184,305	[32]	http://www.cardiogramplusc4d.org/downloads/
Type-II Diabetes	T2D	Metabolic	110,452	[33]	http://diagram-consortium.org/downloads.html
Age-related Macular Degeneration	AMD	Eye	33,976	[34]	http://csg.sph.umich.edu/abecasis/public/amd2015/
Primary Angle Closure Glaucoma	PACG	Eye	26,454	[35]	Journal supplementary material
Femoral Neck Bone Mineral Density	FNBMD	Bone	32,961	[36]	http://www.gefos.org/?q=content/data-release-2012
Lumbar Spine Bone Mineral Density	LSBMD	Bone	31,800	[36]	http://www.gefos.org/?q=content/data-release-2012

* Sample size may be slightly different from that reported in the reference due to technical reasons (e.g. inconsistencies between data sources, different sample sizes for secondary phenotypes).

1. Perry, J.R., et al., *Parent-of-origin-specific allelic associations among 106 genomic loci for age at menarche.* Nature, 2014. **514**(7520): p. 92-7.
2. Day, F.R., et al., *Large-scale genomic analyses link reproductive aging to hypothalamic signaling, breast cancer susceptibility and BRCA1-mediated DNA repair.* Nat Genet, 2015. **47**(11): p. 1294-303.
3. Moffatt, M.F., et al., *A large-scale, consortium-based genomewide association study of asthma.* N Engl J Med, 2010. **363**(13): p. 1211-21.
4. Paternoster, L., et al., *Multi-ancestry genome-wide association study of 21,000 cases and 95,000 controls identifies new risk loci for atopic dermatitis.* Nat Genet, 2015. **47**(12): p. 1449-56.
5. Dubois, P.C., et al., *Multiple common variants for celiac disease influencing immune gene expression.* Nat Genet, 2010. **42**(4): p. 295-302.
6. Franke, A., et al., *Genome-wide meta-analysis increases to 71 the number of confirmed Crohn's disease susceptibility loci.* Nat Genet, 2010. **42**(12): p. 1118-25.
7. Anderson, C.A., et al., *Meta-analysis identifies 29 additional ulcerative colitis risk loci, increasing the number of confirmed associations to 47.* Nat Genet, 2011. **43**(3): p. 246-52.
8. Cordell, H.J., et al., *International genome-wide meta-analysis identifies new primary biliary cirrhosis risk loci and targetable pathogenic pathways.* Nat Commun, 2015. **6**: p. 8019.
9. Okada, Y., et al., *Genetics of rheumatoid arthritis contributes to biology and drug discovery.* Nature, 2014. **506**(7488): p. 376-81.
10. Bentham, J., et al., *Genetic association analyses implicate aberrant regulation of innate and adaptive immunity genes in the pathogenesis of systemic lupus erythematosus.* Nature genetics, 2015.
11. Sawcer, S., et al., *Genetic risk and a primary role for cell-mediated immune mechanisms in multiple sclerosis.* Nature, 2011. **476**(7359): p. 214-9.
12. Consortium, T.I.L.A.E., *Genetic determinants of common epilepsies: a meta-analysis of genome-wide association studies.* The Lancet. Neurology, 2014. **13**(9): p. 893.
13. Jones, S.E., et al., *Genome-wide association analyses in 128,266 individuals identifies new morningness and sleep duration loci.* PLoS Genet, 2016. **12**(8): p. e1006125.
14. Okbay, A., et al., *Genetic variants associated with subjective well-being, depressive symptoms, and neuroticism identified through genome-wide analyses.* Nature genetics, 2016.
15. Rietveld, C.A., et al., *Common genetic variants associated with cognitive performance identified using the proxy-phenotype method.* Proceedings of the National Academy of Sciences, 2014. **111**(38): p. 13790-13794.
16. Okbay, A., et al., *Genome-wide association study identifies 74 loci associated with educational attainment.* Nature, 2016. **533**(7604): p. 539-42.
17. Ripke, S., et al., *Biological insights from 108 schizophrenia-associated genetic loci.* Nature, 2014. **511**(7510): p. 421.
18. Group, P.G.C.B.D.W., *Large-scale genome-wide association analysis of bipolar disorder identifies a new susceptibility locus near ODZ4.* Nature genetics, 2011. **43**(10): p. 977-983.
19. Ripke, S., et al., *A mega-analysis of genome-wide association studies for major depressive disorder.* Mol Psychiatry, 2013. **18**(4): p. 497-511.

20. Neale, B.M., et al., *Meta-analysis of genome-wide association studies of attention-deficit/hyperactivity disorder.* J Am Acad Child Adolesc Psychiatry, 2010. **49**(9): p. 884-97.
21. Boraska, V., et al., *A genome-wide association study of anorexia nervosa.* Mol Psychiatry, 2014. **19**(10): p. 1085-94.
22. Otowa, T., et al., *Meta-analysis of genome-wide association studies of anxiety disorders.* Molecular psychiatry, 2016.
23. Tobacco and G. Consortium, *Genome-wide meta-analyses identify multiple loci associated with smoking behavior.* Nature genetics, 2010. **42**(5): p. 441-447.
24. Locke, A.E., et al., *Genetic studies of body mass index yield new insights for obesity biology.* Nature, 2015. **518**(7538): p. 197-206.
25. Wood, A.R., et al., *Defining the role of common variation in the genomic and biological architecture of adult human height.* Nat Genet, 2014. **46**(11): p. 1173-86.
26. Shungin, D., et al., *New genetic loci link adipose and insulin biology to body fat distribution.* Nature, 2015. **518**(7538): p. 187-96.
27. Ehret, G.B., et al., *The genetics of blood pressure regulation and its target organs from association studies in 342,415 individuals.* Nat Genet, 2016. **advance online publication**.
28. Horikoshi, M., et al., *Genome-wide associations for birth weight and correlations with adult disease.* Nature, 2016. **advance online publication**.
29. Manning, A.K., et al., *A genome-wide approach accounting for body mass index identifies genetic variants influencing fasting glycemic traits and insulin resistance.* Nature genetics, 2012. **44**(6): p. 659-669.
30. Soranzo, N., et al., *Common variants at 10 genomic loci influence hemoglobin A1C levels via glycemic and nonglycemic pathways.* Diabetes, 2010. **59**(12): p. 3229-3239.
31. Teslovich, T.M., et al., *Biological, clinical and population relevance of 95 loci for blood lipids.* Nature, 2010. **466**(7307): p. 707-713.
32. Consortium, C.D., *A comprehensive 1000 Genomes-based genome-wide association meta-analysis of coronary artery disease.* Nature genetics, 2015.
33. Consortium, D.S.D., et al., *Genome-wide trans-ancestry meta-analysis provides insight into the genetic architecture of type 2 diabetes susceptibility.* Nature genetics, 2014. **46**(3): p. 234-244.
34. Fritsche, L.G., et al., *A large genome-wide association study of age-related macular degeneration highlights contributions of rare and common variants.* Nature genetics, 2016. **48**(2): p. 134-143.
35. Khor, C.C., et al., *Genome-wide association study identifies five new susceptibility loci for primary angle closure glaucoma.* Nature genetics, 2016. **48**(5): p. 556-562.
36. Estrada, K., et al., *Genome-wide meta-analysis identifies 56 bone mineral density loci and reveals 14 loci associated with risk of fracture.* Nature genetics, 2012. **44**(5): p. 491-501.
