# Supplementary material for: Systematic tissue-specific functional annotation of the human genome highlights immune-related DNA elements for late-onset Alzheimer’s disease
Source: PLoS Genet. 2017 Jul 24;13(7):e1006933. doi: 10.1371/journal.pgen.1006933 (PMC5546707; doi:10.1371/journal.pgen.1006933)
Supplement: S9 Table — (PDF) [file pgen.1006933.s013.pdf]

S8 Table. Candidate loci for pleiotropic effect between AD and PD.

Chr	Start	End	Gene*	Ref. **
1	89,400,000	89,800,000	*GBP1*	
3	71,600,000	72,000,000	*PROK2*	[1]
3	143,400,000	143,800,000	*SLC9A9*	[2-7]
4	77,000,000	77,400,000	*CCDC158*	[8]
5	10,400,000	10,800,000	*ANKRD33B*	[9]
5	139,400,000	139,800,000	*HBEGF*	[10, 11]
6	27,000,000	27,400,000	*PRSS16*	[12]
6	31,000,000	31,400,000	*HLA-B*	Well studied immune-associated region
6	32,300,000	32,700,000	*HLA-DQB1*	Well studied immune-associated region
6	106,800,000	107,200,000	*AIM1*	[13-16]
9	79,000,000	79,400,000	*PRUNE2*	[17, 18]
16	200,000	600,000	*AXIN1*	[19]
16	30,800,000	31,400,000	*PRSS8*	[8]
17	43,600,000	44,400,000	*MAPT*	[8, 10, 20]
19	52,200,000	52,600,000	*ZNF649*	[21]

* Each locus may contain multiple genes.

** References for reported associations with neurodegenerative diseases or other related traits.

1. Severini, C., et al., *Bv8/prokineticin 2 is involved in Aβ-induced neurotoxicity.* Scientific reports, 2015. **5**.
2. Martinelli-Boneschi, F., et al., *Pharmacogenomics in Alzheimer's disease: a genome-wide association study of response to cholinesterase inhibitors.* Neurobiology of aging, 2013. **34**(6): p. 1711. e7-1711. e13.
3. Esposito, F., et al., *A pharmacogenetic study implicates SLC9a9 in multiple sclerosis disease activity.* Annals of neurology, 2015. **78**(1): p. 115-127.
4. Turner, S.T., et al., *Genomic association analysis identifies multiple loci influencing antihypertensive response to an angiotensin II receptor blocker.* Hypertension, 2012. **59**(6): p. 1204-1211.
5. Mick, E., et al., *Family-based genome-wide association scan of attention-deficit/hyperactivity disorder.* Journal of the American Academy of Child & Adolescent Psychiatry, 2010. **49**(9): p. 898-905. e3.
6. Kondapalli, K.C., et al., *Functional evaluation of autism-associated mutations in NHE9.* Nature communications, 2013. **4**.
7. Chalasani, N., et al., *Genome-wide association study identifies variants associated with histologic features of nonalcoholic fatty liver disease.* Gastroenterology, 2010. **139**(5): p. 1567-1576. e6.
8. Nalls, M.A., et al., *Large-scale meta-analysis of genome-wide association data identifies six new risk loci for Parkinson's disease.* Nature genetics, 2014. **46**(9): p. 989-993.
9. Liu, J.Z., et al., *Association analyses identify 38 susceptibility loci for inflammatory bowel disease and highlight shared genetic risk across populations.* Nature genetics, 2015. **47**(9): p. 979-986.
10. Jun, G., et al., *A novel Alzheimer disease locus located near the gene encoding tau protein.* Molecular psychiatry, 2015.
11. Ashok, A., et al., *Chronic cerebral hypoperfusion-induced impairment of Aβ clearance requires HB-EGF-dependent sequential activation of HIF1α and MMP9.* Neurobiology of Disease, 2016. **95**: p. 179-193.
12. Purcell, S.M., et al., *Common polygenic variation contributes to risk of schizophrenia and bipolar disorder.* Nature, 2009. **460**(7256): p. 748-752.
13. Matarín, M., et al., *A genome-wide genotyping study in patients with ischaemic stroke: initial analysis and data release.* The Lancet Neurology, 2007. **6**(5): p. 414-420.
14. Yashin, A.I., et al., *Joint influence of small-effect genetic variants on human longevity.* Aging, 2010. **2**(9): p. 612-620.
15. Okada, Y., et al., *Genetics of rheumatoid arthritis contributes to biology and drug discovery.* Nature, 2014. **506**(7488): p. 376-81.
16. Martin, J.-E., et al., *A systemic sclerosis and systemic lupus erythematosus pan-meta-GWAS reveals new shared susceptibility loci.* Human molecular genetics, 2013. **22**(19): p. 4021-4029.
17. Hart, A.B., et al., *Genome-wide association study of d-amphetamine response in healthy volunteers identifies putative associations, including cadherin 13 (CDH13).* PloS one, 2012. **7**(8): p. e42646.
18. Potkin, S.G., et al., *Hippocampal atrophy as a quantitative trait in a genome-wide association study identifying novel susceptibility genes for Alzheimer's disease.* PloS one, 2009. **4**(8): p. e6501.

19. Stoothoff, W.H., et al., *Axin negatively affects tau phosphorylation by glycogen synthase kinase 3β.* Journal of neurochemistry, 2002. **83**(4): p. 904-913.
20. Lei, P., et al., *Tau protein: relevance to Parkinson's disease.* The international journal of biochemistry & cell biology, 2010. **42**(11): p. 1775-1778.
21. Winham, S., et al., *Genome-wide association study of bipolar disorder accounting for effect of body mass index identifies a new risk allele in TCF7L2.* Molecular psychiatry, 2014. **19**(9): p. 1010-1016.
